# The Relationship between Gene Isoform Multiplicity, Number of Exons and Protein Divergence

**DOI:** 10.1371/journal.pone.0072742

**Published:** 2013-08-30

**Authors:** Jordi Morata, Santi Béjar, David Talavera, Casandra Riera, Sergio Lois, Gemma Mas de Xaxars, Xavier de la Cruz

**Affiliations:** 1 Department of Structural Biology, Institut de Biologia Molecular de Barcelona (IBMB)-Consejo Superior de Investigaciones Científicas (CSIC), Barcelona, Spain; 2 Faculty of Life Sciences, Manchester University, Manchester, United Kingdom; 3 Laboratory of Translational Bioinformatics in Neuroscience, Vall d'Hebron Institute of Research (VHIR), Barcelona, Spain; 4 Laboratori de Botànica, Facultat de Farmàcia, Universitat de Barcelona, Barcelona, Spain; 5 Institució Catalana de Recerca i Estudis Avançats (ICREA), Barcelona, Spain; Tel Aviv University, Israel

## Abstract

At present we know that phenotypic differences between organisms arise from a variety of sources, like protein sequence divergence, regulatory sequence divergence, alternative splicing, etc. However, we do not have yet a complete view of how these sources are related. Here we address this problem, studying the relationship between protein divergence and the ability of genes to express multiple isoforms. We used three genome-wide datasets of human-mouse orthologs to study the relationship between isoform multiplicity co-occurrence between orthologs (the fact that two orthologs have more than one isoform) and protein divergence. In all cases our results showed that there was a monotonic dependence between these two properties. We could explain this relationship in terms of a more fundamental one, between exon number of the largest isoform and protein divergence. We found that this last relationship was present, although with variations, in other species (chimpanzee, cow, rat, chicken, zebrafish and fruit fly). In summary, we have identified a relationship between protein divergence and isoform multiplicity co-occurrence and explained its origin in terms of a simple gene-level property. Finally, we discuss the biological implications of these findings for our understanding of inter-species phenotypic differences.

## Introduction

**Figure 1:**
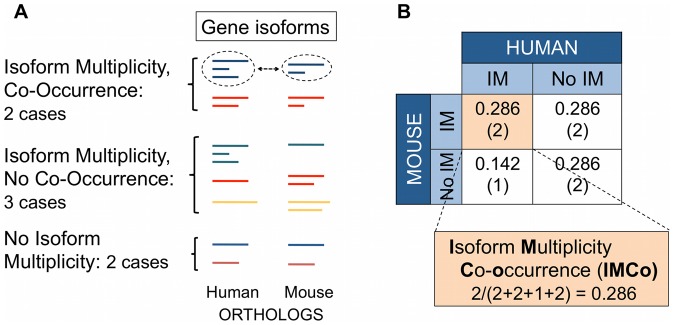
Description of isoform multiplicity co-occurrence (IMco). The figure illustrates how IMco is computed. We start from a set of human-mouse orthologs for which isoform annotations are available in ENSEMBL (or VEGA), as shown in (A). We will say that one gene has isoform multiplicity (IM) when it has more than one isoform. The scheme shows different instances that cover all possible IM combinations for human-mouse ortholog pairs: both orthologs have IM, only one has IM, and no ortholog has IM. (B) IMco will correspond to the fraction of cases for which both orthologs have IM; it is computed as shown in the table.

Understanding the molecular basis of phenotypic differences (PheDif) between organisms is a fundamental problem in modern biology. A large body of evidence shows that changes in phenotype can arise from gene regulation and protein divergence [1]–[3]. Recent data show that this is also the case for other processes, particularly those leading to gene isoform multiplicity (IM), such as alternative splicing or alternative translation initiation and/or termination [1], [2], [4]–[7]. However, and in spite of its relevance, we still know little about the interplay between all these processes and how it leads to PheDif between species [1], [3]. In the present work we address this problem in the case of human and mouse, focusing on the relationship between protein divergence and isoform multiplicity co-occurrence (IMco) (Figure 1).

IM, a gene property reflecting the fact that genes can express more than one isoform, can result from alternative splicing of pre-mRNA or alternative translation initiation and/or termination, although it is generally accepted that alternative splicing is the most important source [8]. Alternative splicing has such a potential to sample protein function space [8]–[10] that it has been postulated as an important contributor to complexity differences between organisms [11], [12]. The idea that variations in alternative splicing patterns play such a role is supported by a large number of studies relating alternative splicing and disease [13]–[15]. Also, at a more general level, this idea has been explored and tested by many researchers who have looked at differences on the amount of alternative splicing between organisms [5], checked the conservation of alternative splicing events between species [7], [16]–[28] and of AS structural/functional impact [29], explored the role of premature termination codons [30], differential regulation [31], etc. The results of these studies indeed point to a clear relationship between interspecies differences and the distribution of alternative splicing properties.

Mechanistically different from alternative splicing, but related to it, alternative transcription initiation and termination are two additional sources of multiple gene isoforms [6], [32]. They can result in proteins with different N- or C-terminal ends, or more substantial sequence changes [6], [32], and may have functional properties similar to those of alternative splicing isoforms, e.g. behave as dominant-negatives or have new cellular locations [32]. Recent data indicate that the relevance of these mechanisms may be comparable to that of AS, and that both phenomena are tightly related [6], [33].

As mentioned before, we still do not know the relationship between protein divergence and IM. Are they independent? Do they covary? And if so, in which manner? Answering these questions would constitute an important advance towards understanding the molecular basis of inter-species PheDif. Here we address this problem by studying how IM co-occurrence (IMco) between human-mouse orthologs (Figure 1; see *Materials and Methods*) varies as a function of protein divergence (PD). Note that instead of using IM co-occurrence we could have used another IM-related property, like isoform number or nature, or conservation of IM signals at the gene level. However, there is an already large number of studies addressing these problems [7], [16]–[29], [34]–[41].

We have divided our work in three parts. First, description of the IMco-PD relationship for the human-mouse case. Second, explanation of this relationship in terms of a more fundamental relationship, between number of exons of the largest isoform and PD. Third, given the interest of this last relationship, we explored whether it was present in other species.

**Figure 2:**
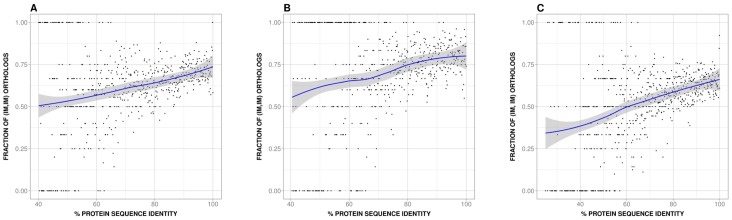
Relationship between isoform multiplicity co-occurrence (IMco) and protein divergence (PD). In (A), (B) and (C) we plot IMco (the fraction of (IM, IM) ortholog pairs, see Figure 1 and *Materials and Methods*) as a function of PD (measured using the percentage of sequence identity). Black dots are used to display the raw, unprocessed data; a blue line is used for the smoothed data and in grey we show the envelope (see *Results* section). In both cases we can see that there is a monotonically increasing relationship between IMco and PD (see text). Outliers from this trend define two lines, one at 0 and the other at 1; these outliers result from IMco estimates obtained with less than 5 observations. They essentially disappear when eliminating these poorer estimates (Figure S1). (A) and (B) where obtained with a Uniprot/SwissProt/RefSeq-based sequence dataset and Ensembl and VEGA isoform annotations, respectively; (C) was obtained using Ensembl data for both genes and transcripts. The monotonic trend is comparable in the three figures, although in (C) the curve shows a slight shift towards lower IMco values resulting from lower amount of genes with IM.

Our results show (Figure 2) that IMco and PD are monotonically related for human-mouse orthologs. This observation could be rationalized combining two facts: (i) the connection between IM and the number of exons of the largest isoform, a fact expected from previous work [42]–[44]; and the relationship between number of exons of the largest isoform and PD, an unexpected fact. Finally, we find that this second relationship is also present in other species, although with some variations (the most extreme being for fruit fly, for which the monotonic trend was reversed). In the Discussion section, we first explore the origin of these relationships; then, for the human-mouse case, we propose how the IMco-PD relationship can be used to understand the relative contribution of PD and IMco to the generation of PheDif between these species.

## Materials and Methods

### The Annotated Ortholog Datasets

In this work we have used ortholog sets to study the IMco-PD relationship in human-mouse, to trace its origin, and to explore the relationship between number of exons and PD in different species. We therefore had to obtain a set of gene orthologs for each human-species case, as well as transcript annotations for all the genes. We describe below how this was done. Note that we treat separately the human-mouse case, which constituted the core of this work, because more data were available for its study.

#### 1. Human-mouse

We produced three datasets of annotated orthologs, to ensure the robustness of our results.

Datasets 1 and 2. Here the orthology relationships were obtained with InParanoid [45], using as starting point a set of human and mouse sequences obtained by combining data from two manually curated databases, UniProt/SwissProt [46], [47] and RefSeq [48], following a five-step protocol (we explain it for human only, the same steps were applied to mouse). **First**, we obtained all human genes from UniProt/SwissProt (release 54.7) and RefSeq (release 27). **Second**, we established the equivalence between the two versions of the same gene using the GeneId identifier. **Third,** we eliminated those genes for which sequence conflicts were more than expected by random; this was done computing the frequency of sequence conflicts from UniProt/SwissProt sequence data, and using it in a Poisson model for the distribution of sequence conflicts (analogous to that described in Altmann et al. [49]) to exclude those genes for which the number of conflicts had a probability lower than 0.05 (this was done for UniProt/SwissProt data only). **Fourth,** we eliminated those isoforms for which the RefSeq record started by either XP_ or ZP_. **Fifth,** from the final set of human and mouse genes we eliminated those cases for which no correspondence could be established between UniProt/SwissProt and Ensembl records (this was required to use isoform annotations from Ensembl).

We used the longest isoform for each gene to find the orthology relationships between human and mouse. We eliminated those cases for which InParanoid [45] found no orthologs in one of the species, or for which no unique human-mouse orthology relationship was available. At the end of this process we obtained a set of 11969 pairs of human-mouse orthologs that was the starting point for the subsequent analyses.

Transcript annotations for the genes in this list defined the differences between dataset 1 and 2. For dataset 1 they were obtained from Ensembl [50] (version 65, GRCh37.p5 and NCBIM37 for human and mouse respectively); for dataset 2 they were obtained from VEGA [51] (version 45, GRCh37.p5 and NCBIM37 for human and mouse respectively), without applying any filtering in neither case.

In summary, we obtained two annotated sets human-mouse orthologs (11969 pairs for dataset 1 and 6795 pairs for dataset 2, numbers resulting from matching Uniprot/SwissProt records and Ensembl and VEGA records, respectively), with a fraction of IM of: 86% (human) and 72% (mouse), dataset 1; and 85% (human) and 84% (mouse), dataset 2. These datasets were used to produce the results in Figures 2A (dataset 1) and 2B (dataset 2).

Dataset 3. The list of human-mouse orthologs was obtained with EnsemblCompara [52]. We used only Ensembl (version 69) data for genes and transcripts, imposing that both had to be labelled as KNOWN.

In summary, we obtained one annotated set of 15134 human-mouse ortholog pairs, with a fraction of IM of 80% (human) and 54% (mouse). This dataset was used to produce the results in Figure 2C.

#### 2. IM-annotated gene sets for other species

For a set of 6 species (chimpanzee, cow, rat, chicken, zebrafish and fruit fly) we characterized the relationship between number of exons of the longest species and PD. We used only Ensembl (version 69) [50] data for genes and transcripts, additionally imposing that both had to be labelled as KNOWN. The lists of orthologs were obtained with the standard program, EnsemblCompara [52]. The statistics of the resulting datasets are summarized in Table 1.

**Table 1:** Statistics of the datasets used in this work: number of ortholog pairs and percentage of genes with IM (the latter refers only to the model species, not to human).

Species	Human Chimpanzee	Human Cow	Human Mouse	Human Rat	Human Chicken	Human Zebrafish	Human Fruit fly
Number of ortholog pairs	15954	10790	15134	14150	4238	6714	3108
% genes with IM (non-human species)	5.0	8.5	54.2	27.4	37.1	34.0	31.0

In all cases, including human and mouse, exon annotations were obtained from Ensembl.

### IM co-occurrence (IMco)

IMco, explained in Figure 1, was defined as the fraction of pairs for which both orthologs had more than one isoform. We represented this fraction as a function of the percentage of sequence identity between ortholog proteins, which is a measure of PD (see section “Computation of PD” below). IMco can also be expressed in a more formal view as a probability, P(IM_H_, IM_M_|x) (Note: to express joint events we use commas instead of ∩, as in Bishop [53], e.g.):
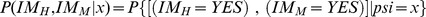
(1)where IM_H_ is an indicator variable with two values: YES, when human orthologs have more than one isoform, and NO, when they only have one. IM_M_ is the mouse equivalent of IM_H_. psi is a variable that corresponds to protein sequence identity, and x to its actual value. Note: here we use the human-mouse case to explain IMco, but an exactly analogous definition applies to other cases.

In summary, IMco is a similarity measure between sets of human-mouse paired orthologs. It varies between 0 and 1; 0 happens when there is no ortholog pair with IM for both genes, 1 happens when all ortholog pairs have IM for both genes.

Using basic probability results [54] we can express P(IM_H_, IM_M_|x) (Equation 1) in terms of the product of the two species-specific IM, P(IM_H_|x) and P(IM_M_|x):
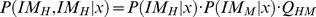
(2)where Q_HM_ = {[P(IM_H_|IM_M_,x)⋅P(IM_M_|IM_H_,x)]/[P(IM_H_|x)⋅P(IM_M_|x)]}^1/2^. If the contribution of IM_H_ and IM_M_ to IMco is independent, then Q_HM_ = 1 and P(IM_H_, IM_M_|x) = P(IM_H_|x)⋅P(IM_M_|x). We used Equation 2 as a starting point to explore the relationship between P(IM_H_, IM_M_|x) and gene properties.

### Computation of PD

As a measure of PD we used the percentage of sequence identity between ortholog proteins. It was computed after global sequence alignment (using a standard dynamic programming algorithm [55]) of the longest isoform from each species, and was equal to the number of identical residue pairs (n_id_) in the alignment divided by the average of the human (n_human_) and mouse (n_mouse_) protein lengths: 2.n_id_/(n_human_+n_mouse_). To avoid any confusion in the interpretation of our results, it must be noted that high sequence identities correspond to low PD, and low sequence identities to high PD.

The longest isoform was used for consistency with Inparanoid [45] and EnsemblCompara [52] orthology computations, where it is also used. The sequence of the longest isoform has also been used in different works, e.g. in evolutionary studies of genomic duplication [56] and of mammalian gene families [57], in gene morbidity classification [58], etc.

### Statistical Computations

Statistical computations were carried with R [59]. For illustration purposes, in most of the figures we include the smoothed version of the data, with a shaded envelope. This representation was also obtained with R; it corresponds to a LOESS smoothing, where the envelope reflects data dispersion (its size is inversely associated to sample size: the more sample, the tighter the envelope).

### Mouse IM Annotations from RNA-seq Data

RNA-seq data for mouse [28] were retrieved from NCBI's GEO database [60]: accession number GSE41637. We processed these data to obtain IM annotations for the mouse genome using Cuffcompare [61], with Ensembl as a reference and default parameters. After parsing the resulting output we got a list of genes with their isoforms, according to the RNA-seq experiment. These annotations were obtained for each tissue sample and individual. To carry our analysis, we first collapsed IM annotations from different tissues of an individual as follows: a gene was annotated as having IM when it had IM in at least one tissue. Annotations from the three individuals were subsequently collapsed in two different ways: a gene was annotated as having IM when (i) it had IM in at least one individual or (ii) in each individual.

## Results

In the following we describe the three parts of our work: (i) the description of the relationship between IMco (Figure 1) and PD in human-mouse orthologs; (ii) the explanation of this relationship in terms of another, more fundamental relationship, between number of exons of the longest isoform and PD; and (iii) identification of this second relationship in other species (chimpanzee, rat, cow, chicken, zebrafish and fruit fly). The first two parts are presented in section “A. The human-mouse case”, and the third part in section “B. The relationship between number of exons of the largest isoform and PD in other species”.

Note 1: as explained before, we define IMco as the fraction of human-mouse ortholog pairs for which both genes have more than one isoform (Figure 1, and Equation 1 in *Materials and Methods*). For simplicity, sometimes we will also refer to IMco as P(IM_H_, IM_M_|x), and to species-specific IM (the fraction of genes with IM in one species) as P(IM_H_|x) and P(IM_M_|x), for human and mouse, respectively. In all cases x is the percentage protein sequence identity, used as a measure of PD (see *Materials and Methods*).

Note 2: as explained before we measure PD using percentage of protein sequence identity (see *Materials and Methods*). In the text, sometimes we will refer to PD, sometimes to sequence identity (mostly when describing the figures). The global meaning will be the same, with one subtle difference that appears when considering monotonic trends. Because low PD corresponds to high sequence identities, and high PD corresponds to low sequence identities, an increasing monotonic trend involving sequence identity will correspond to a decreasing monotonic trend involving PD.

### A. The Human-mouse Case

This case was treated separately because the genes of these two species are more extensively annotated in the databases used (Ensembl, VEGA and UniProt/SwissProt, RefSeq).

#### A.1. The IMco-PD relationship

As mentioned in the *Materials and Methods* section, we used three different datasets to obtain the IMco-PD relationship and check its robustness. We start the section with the results for datasets 1 and 2, for which sequences came from UniProt/SwissProt-RefSEq, orthology relationships from InParanoid and transcript annotations from either Ensembl (dataset 1) or VEGA (dataset 2). We finish the section with the results for dataset 3, for which all the sequence and transcript data came from Ensembl and the orthology relationships from EnsemblCompara.

For dataset 1, when plotting IMco against percentage of protein sequence indentity (Figure 2A) we observed a monotonically increasing (decreasing, if we think in terms of PD) relationship between these two variables (Spearman's rank correlation = 0.3, p-value = 10^−12^. Note: in the following we will use rho to refer to Spearman's rank correlation). Simply stated, we observed that as sequence identity grows (or PD decreases) it is easier to find ortholog pairs where both genes have IM. It has to be noted that IMco was estimated from the data available at each individual value of sequence identity, no data clustering was applied. At low sequence identities, because there were less human-mouse ortholog pairs IMco estimates were noisier. In the most extreme case, only one ortholog pair was available, thus leading to outlier points populating the two extreme IMco values, 0 and 1, for sequence identity below 60%–70% (Figure 2A). To correct for this sample effect, we followed two different approaches. In the first one, we computed the smoothed version of the data (see *Materials and Methods*), where this effect is alleviated. The resulting curve (continuous line in Figure 2A) confirmed the monotonic trend found using raw data. In the second approach we discarded, from the original dataset, any IMco estimate obtained with less than 5 observations. The results (Figure S1A) show the existence of the monotonically increasing relationship with a correlation higher than for the non-pruned, original dataset (rho = 0.43, p-value∼10^−21^); for completeness, we also applied the smoothing procedure to this filtered dataset with essentially the same result (Figure S1A).

Next, we reproduced our analyses with dataset 2, finding the same monotonic relationship between IMco and sequence identity (Figure 2B; rho = 0.12, p-value∼0.004). Here the sampling problem was more severe, as the number of ortholog pairs (6795) was smaller than for the dataset 1 (11969). Application of the smoothing procedure (Figure 2B) supported the existence of the monotonically increasing relationship. This was also confirmed when plotting IMco vs. sequence identity, after eliminating from the original dataset those IMco estimates obtained with less than 5 observations (Figure S1B; rho = 0.35, p-value∼10^−11^). Again, application, for completeness, to this filtered dataset of the smoothing procedure gave the monotonic relationship (Figure S1B).

Finally, we reproduced the previous results with dataset 3. As can be seen in Figure 2C, we observe again the monotonic relationship between IMco and sequence identity (rho = 0.4; p-value∼10^−22^). This is relevant because we used this simpler data retrieving protocol for the other cases studied (see section “B. The relationship between number of exons of the largest isoform and PD in other species” below).

### A.2. Understanding the Gene-level Origin of the IMco-PD Relationship: the Connection Between Number of Exons and PD

**Figure 3:**
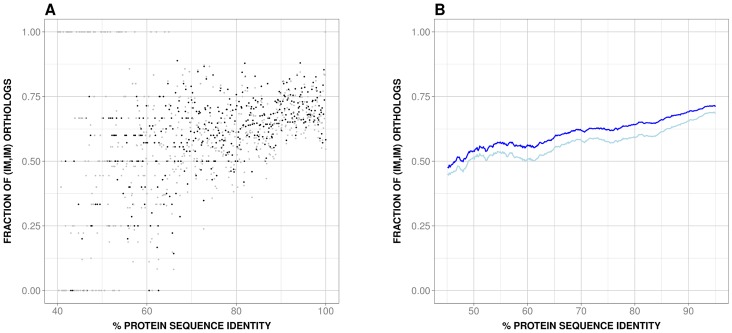
The contribution of species-specific isoform multiplicity (IM) to isoform multiplicity co-occurrence (IMco). Here we compare IMco with P(IM_H_|x)⋅P(IM_M_|x), the product of species-specific IM and a term of IMco, as shown in Equation 2 (see *Materials and Methods*). In (A) we show the raw data representation: we can observe an important overlap between both data clouds, as well as a similar monotonic trend, something confirmed in (B) where we show the smoothed data. In (A) we used black and grey for IMco and P(IM_H_|x)⋅P(IM_M_|x), respectively; in (B) we used dark and light blue, respectively.

This explanatory analysis was carried using datasets 1 and 2. As mentioned in the *Materials and Methods* section, IMco depends on the product of species-specific IM (Equation 2). This could be clearly seen when comparing P(IM_H_, IM_M_|x) (that is, IMco) and P(IM_H_|x)⋅P(IM_M_|x) for dataset 1: using raw data we observed (Figure 3A) an important overlap between the corresponding data clouds. This similarity was more clearly seen when plotting the smoothed versions of P(IM_H_|x)⋅P(IM_M_|x) and P(IM_H_, IM_M_|x): as expected, the monotonically increasing behavior of the resulting curves was essentially the same (Figure 3B); there was only a small shift between both curves. The same result was obtained when using dataset 2 (Figure S2).

**Figure 4:**
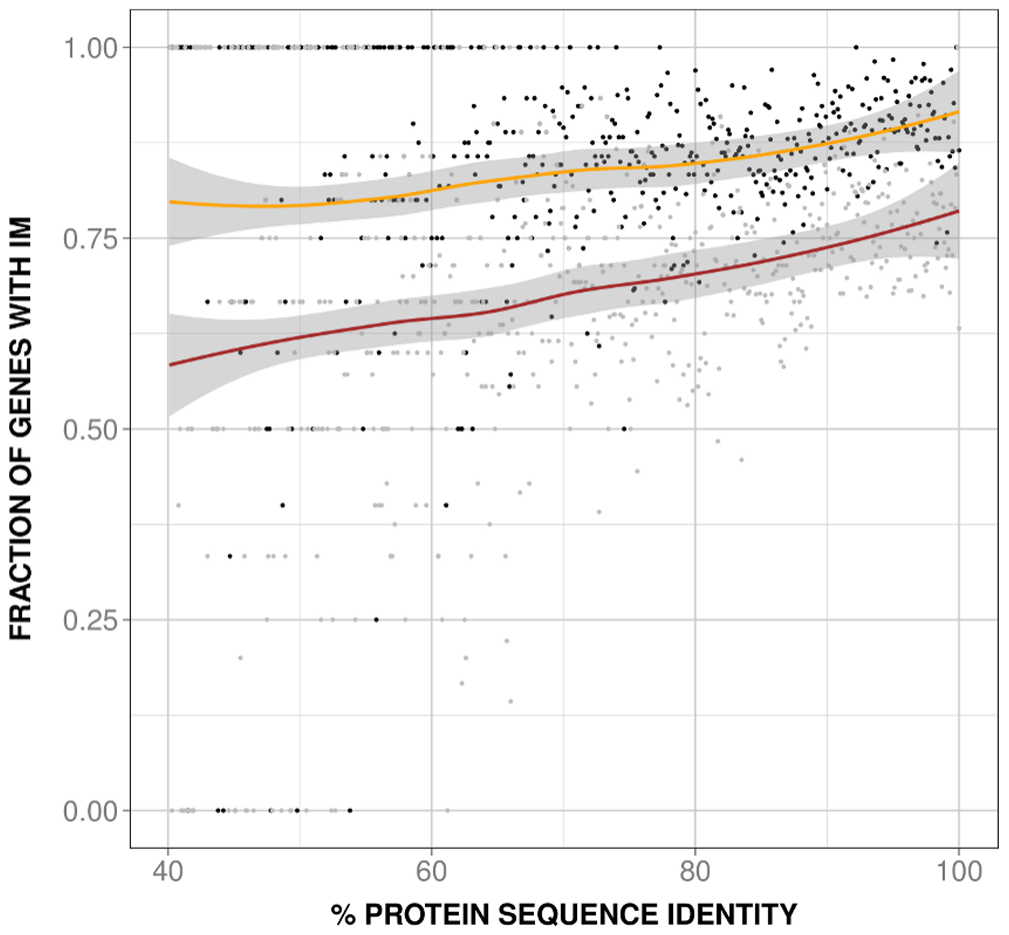
Species-specific isoform mutiplicity (IM) vs. protein divergence (PD). Here we show the relationship between species-specific IM and PD, for both human and mouse genes. Black and grey dots are used for human and mouse, respectively. We observe the same monotonic trend for both species, a result that provides a simple explanation for the also monotonic behavior of P(IM_H_|x)⋅P(IM_M_|x) (see Figure 3), the product of species-specific IM. In addition, this result is an important intermediate step that will allow us to trace back the result in Figure 2 to a simple gene-level property (see text and Figure 6): the number of exons of the longest gene isoform. The continuous lines represent the smoothed version of the raw data (yellow and red for human and mouse, respectively; grey for the envelope) and lead to the same interpretation.

**Figure 5:**
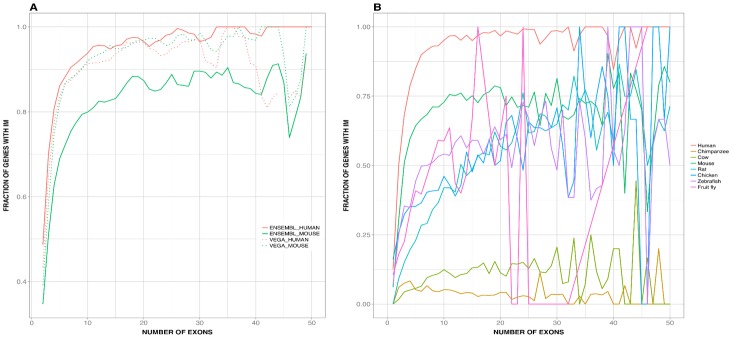
The relationship between isoform multiplicity (IM) and number of exons of the largest isoform. The figure illustrates the relationship between these two properties for both human and mouse genes (A), and for these and other species (B). (A) and (B) differ in the data origin: (A) was obtained using UniProt/SwissProt/RefSeq sequences and Ensembl/VEGA transcript annotations; (B) was obtained using only Ensembl data. In general, we see the same trend: an increasing monotonic relationship which approaches 1 asymptotically, indicating that the larger the number of exons of the gene, the larger the number of isoforms of this gene. The fluctuations observed are due to a combination of factors: irregular isoform annotations (e.g. fruit fly), or low sample effects (particularly, for number of exons>30). Chimpanzee is an exception due to a very low percentage of transcript annotations (only 5% of the genes had multiple isoforms in the version of Ensembl used).

**Figure 6:**
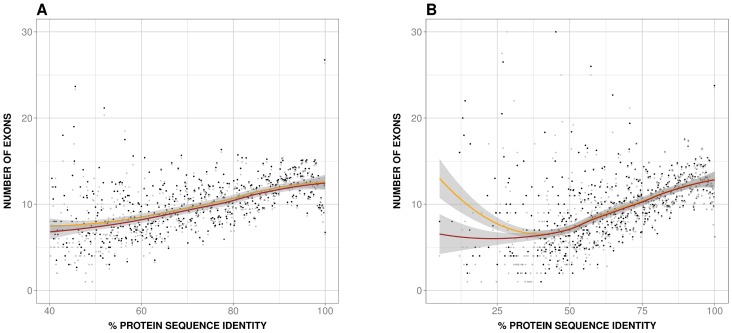
Number of exons vs. protein divergence (PD). Here we show the relationship between number of exons of the largest isoform and PD, for both human and mouse genes. Black and grey dots are used for human and mouse, respectively. We observe the same monotonically increasing trend for both species, which provides a natural explanation for the behavior of species-specific IM seen in Figure 4. The smoothed version is shown with a continuous line (yellow and red for human and mouse, respectively; grey for the envelope). The relevance of this result is that we have identified a simple gene property contributing to the relationship between IM co-occurrence, IMco, and PD shown in Figure 2. (A) was obtained using UniProt/SwissProt/RefSeq sequences and Ensembl transcript annotations; (B) was obtained using only Ensembl data.

On the basis of the previous considerations we decided to study separately the behavior of the human and mouse species-specific IM, P(IM_H_|x) and P(IM_M_|x) respectively, as a function of PD. In both cases we observed a monotonic relationship between species-specific IM and PD (Figure 4), also present when using dataset 2 (Figure S3) and mouse RNA-seq data [28] (Figure S4). Because alternative splicing is a main contributor to IM and depends on the gene's number of exons [42]–[44] (Figure 5), we checked if there was a relationship between sequence identity and number of exons of the largest isoform. We found (Figure 6A; rho = 0.6 and 0.6, p-value∼10^−51^ and ∼10^−58^, for human and mouse, respectively) that this was indeed the case, with larger genes (in terms of number of exons) being more abundant at higher than at lower sequence identities. The same result was obtained with dataset 2 (Figure S5; rho = 0.5 and 0.5, p-value∼ 10^−33^ and ∼10^−38^ for (A); rho = 0.4 and 0.4, p-value∼10^−16^ and ∼10^−17^, (B); for both (A) and (B) rho and p-values are given first for human and then mouse data) and dataset 3 (Figure 6B; rho = 0.6 and 0.6, p-value = ∼10^−59^ and ∼10^−80^, for human and mouse, respectively).

**Figure 7:**
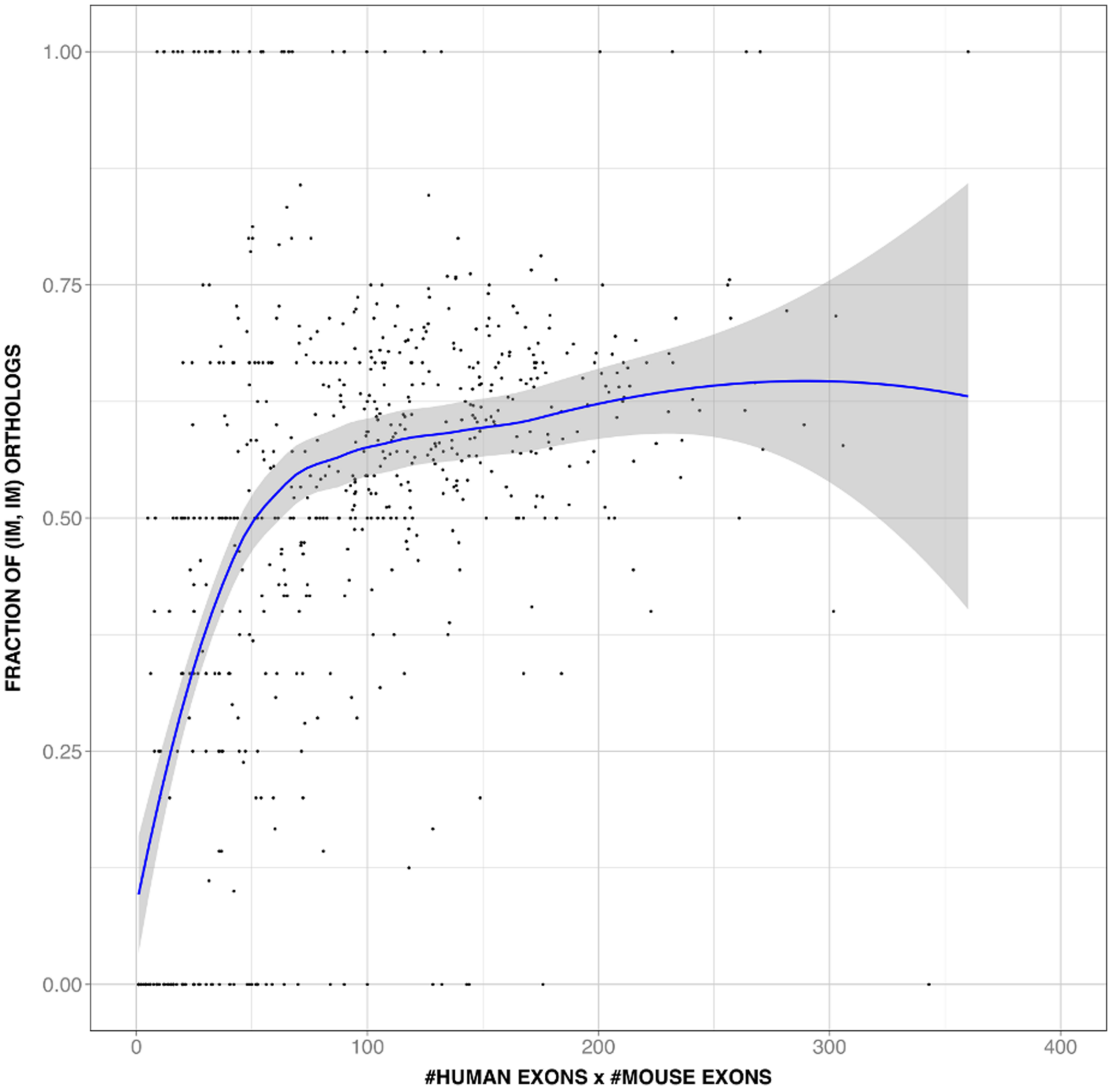
Number of exons is a component of isoform multiplicity co-occurrence (IMco). We display (black dots) the relationship of IMco vs. the product of human and mouse number of exons. We chose the product because it is a priori related to IMco through Equation (2) and Figure 5. The result shows the existence of a monotonically increasing relationship. Shown in blue is the smoothed version of the raw data, and the envelope in grey.

The value of this unexpected relationship lies on two facts. First, it provides a gene-level explanation for the IMco-PD relationship (Figure 2); to add further support to this explanation we showed that IMco depends on the number of exons of the human and mouse longest isoforms (Figure 7; rho = 0.5, p-value∼10^−37^). Second, and particularly if present in other species, this relationship provides a simple way to integrate and understand at the gene level two molecular sources of PheDif such as IM and PD.

### B. The Relationship between Number of Exons of the Largest Isoform and PD in Other Species

**Figure 8:**
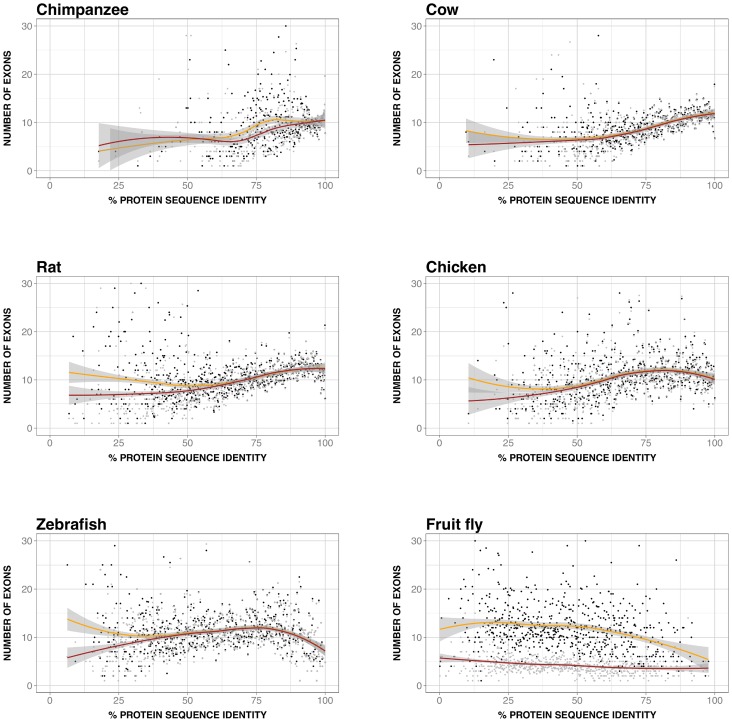
Number of exons vs. protein divergence (PD) in other species. This figure is equivalent to Figure 6, but in this case we show the results for six other species. In each plot we represent the data for human (black dots; smoothed version in yellow) and the other species (grey dots; smoothed version in red); the envelopes of the smoothed versions are shown in grey. From left to right and top to bottom we have the results for: chimpanzee (rho = 0.3 and 0.4, p-value∼10^−10^ and ∼10^−17^, for human and chimpanzee, respectively), cow (rho = 0.6 and 0.6, p-value∼10^−59^ and ∼10^−66^), rat (rho = 0.4 and 0.6, p-value∼10^−25^ and ∼10^−66^), chicken (rho = 0.3 and 0.4, p-value∼10^−21^ and ∼10^−27^), zebrafish (rho = 0.0 and 0.1, p-value∼0.39 and ∼10^−05^) and fruit fly (rho = −0.3 and−0.3, p-value∼10^−14^ and ∼10^−14^). In all cases we observe a nearly monotonic relationship, which is increasing for vertebrates, and decreasing for fruit fly.

Given its interest, we explored if this relationship was present in other species (chimpanzee, cow, rat, chicken, zebrafish, and fruit fly), finding that this was always the case (Figure 8). However, there was a difference between vertebrates and fruit fly. For vertebrates, the curves for all the species had similar features, including a common monotonic behavior in the central sequence identity range (roughly between 50% and 80%–90%) and deviations at the extremes. For fruit fly the relationship was also significant (rho = −0.3, p-value∼10^−14^), but the monotonic trend was opposite to vertebrates (Figure 8).

In summary, our results show that for all the species studied there was a relationship between number of exons of the largest isoform and PD, although its monotonic nature changed from vertebrates to fruit fly.

**Figure 9:**
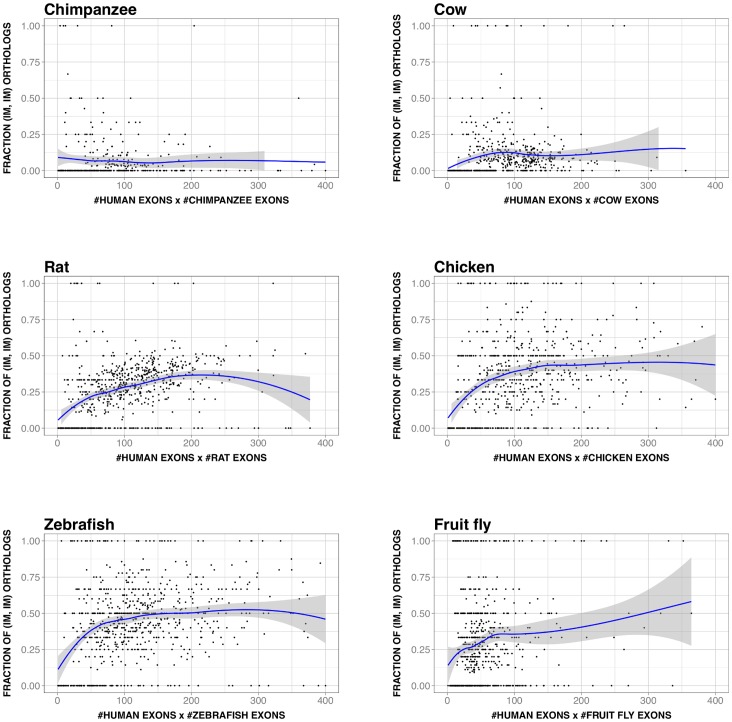
Number of exons is a component of isoform multiplicity co-occurrence (IMco), other species. This figure is equivalent to Figure 7, but in this case we show the results for six other species (raw data with black dots, smoothed curve in blue and envelope in grey). We display the relationship of IMco vs. the product of human and mouse number of exons. We chose the product because it is a priori related to IMco through Equation (2) and Figure 5. From left to right and top to bottom we have the results for: chimpanzee (rho = 0.1, p-value = ∼10^−02^), cow (rho = 0.3, p-value = ∼10^−17^), rat (rho = 0.4, p-value = ∼10^−32^), chicken (rho = 0.3, p-value = ∼10^−19^), zebrafish (rho = 0.3, p-value = ∼10^−14^) and fruit fly (rho = 0.2, p-value = ∼10^−10^). Because the extent of isoform annotations goes from low to very low (only 5% of chimpanzee genes had more than one isoform), the relationships are weaker than in Figure 7.

As before (in Figure 7), we explored to which extent number of exons and IMco were connected. It has to be noted that in this case IMco estimates were subject to a larger error, given that IM annotations were less extensive (Table 1). Nonetheless, our results showed (Figure 9) that number of exons was a component of IMco. In some cases the relationship was weak, something probably due to the following causes: low IM coverage, and/or existence of a bias in the human-animal model ortholog gene set, a bias which could either be of technical (e.g. low IM coverage in chimpanzee) or of biological (e.g. smaller number of orthologs for human-fruit fly) origin.

## Discussion

Recent years have witnessed important advances in the identification of the molecular sources of PheDif between organisms [4], [7], [62]. However, we still do not know how these molecular sources relate [3], [4]. For example, in general we ignore whether and to which extent they cooperate [3], [63], or if there is some degree of equivalence between them (comparable to that proposed for alternative splicing and gene duplication [64], [65]), etc. A series of studies have started to clarify this issue for PD, promoter-level divergence and gene expression [62], [66]–[70]. Here we advance in this direction by analyzing the relationship between IMco and PD (Figure 1).

To relate IMco and PD we first have to take into account that IM can be studied from two different sides: (i) the isoform/signal side, in which isoforms, or their associated gene-level signals, are compared between species; and (ii) the pattern of IM presence/absence between orthologs. An important volume of work has been devoted to the first topic. For example, Modrek and Lee [20] found, using a bioinformatics approach, that exons constitutively spliced or present in major isoforms were conserved between species, while those present in minor isoforms were mainly species-specific. This result was experimentally confirmed and extended by Pan et al. [22], [37] using specifically designed microarrays and splicing prediction methods. Sorek and Ast [71] and Sugnet et al. [72] have focused on the differences in the nature of the flanking intronic sequences between constitutive and alternatively spliced exons; several other aspects of splicing signals and their conservation have been also addressed [25], [34], [36], [38]–[40], [73]. In all these studies gene sequence similarity was related in one way or another to IM conservation between species, thus providing a good basis for understanding the coordinated contribution of these factors to PheDif.

The situation is different when we consider IM from the point of view of its pattern of presence/absence in orthologs. We know that presence of IM implies the introduction of a new regulatory level in gene expression [8]. Therefore, simple switching from IM to noIM between orthologs may imply an important change, even before taking into consideration any possible difference between isoforms. At the genome level, this suggests that differences between organisms in the fraction of genes with IM may contribute to explain their PheDif [5], [11], [74], [75]. While initial tests of this hypothesis led to controversial results [74], [75], it was subsequently shown that IM could indeed play the proposed role [5]. Our work can be seen as a natural extension of this research: here the results of IM co-occurrence are broken down along the PD range, to see what is the relationship between these two variables and whether it can be explained in terms of some gene property.

Using a set of 11969 human-mouse orthologs we found that the IMco-PD relationship was monotonic (Figure 2A), a result confirmed with a second (Figure 2B) and third (Figure 2C) datasets. Overlap between coding region and alternative splicing signals [76]–[80] could provide a possible explanation for the monotonic nature of the relationship. However, comparison of P(IM_H_, IM_M_|x) and P(IM_H_|x)⋅P(IM_M_|x) showed that species-specific IM is a major contributor to this relationship (Figure 3), and that it monotonically depends on PD (Figure 4). Interestingly, a relationship similar to the latter had also been found in different species by Su et al. [65] for duplicated genes. From this point, and with the link between IM and number of exons in mind (Figure 5), we were able to trace back the monotonic behavior of IMco to a relationship between number of exons and PD (Figures 6). This relationship was a priori unexpected, although a related result, involving the size of protein alignments instead of the number of exons, had been described by Makalowski et al. [81] for a small set of human-mouse orthologs. We then decided to check if number of exons and PD were also related in other species, finding that this was the case (Figure 8). Actually, the monotonic behavior was comparable for vertebrates, although inverted for fruit fly.

The connection between number of exons and PD may be used to suggest a biological reason for the origin of the IMco-PD relationship. We know that the number of exons of an isoform is directly related to the length of the resulting protein. Interestingly, a few years ago Lipman et al. [82] found a link between protein conservation and sequence length: conserved proteins tended to be larger than non-conserved ones. Although their definition of conservation was discrete (two classes: conserved and non-conserved) and based on taxonomic considerations, their use of sequence comparisons made it relatively similar to our PD. When replacing sequence length by number of exons, the finding of Lipman et al. [82] qualitatively corresponds to our observed relationship between number of exons and PD (Figure 6). These authors explain their results in terms of a balance between different constraints [82]. Functional constraints would be higher for conserved proteins, therefore limiting any drastic sequence change, like deletions. On the contrary, for non-conserved proteins functional constraints would be weaker, and gradually replaced by the pressure to minimize translation costs. The latter, combined with a higher frequency of sequence deletions over insertions, would result in smaller proteins. This explanation can be naturally transferred to the monotonic dependence between number of exons and PD (Figure 6) and extended, with caution, to the IMco-PD relationship, which would then result from a balance between gene function and protein synthesis costs. Finally, it has to be mentioned that a combination of recent results from two groups [83], [84] suggest the existence of a relationship between number of exons and evolutionary rate consistent with our work.

### Using the PD-IMco Relationship to Improve Our Understanding of the Molecular Basis of Inter-species PheDif

The final goal of integrating the different sources of inter-species PheDif is to reach a better understanding of the molecular basis of these PheDif [4]. Within this context we will discuss the explanatory power of the IMco-PD relationship and what are its present limitations. To this end we need to see how changes in PD and IMco relate to changes in molecular function, something that will require different approaches, as PD is a single-gene-based measure, and IMco is multigene. In the case of PD we know that most features of protein function are conserved, on the average, above a certain sequence identity threshold: 60–70% for enzyme function [85]–[87]; 30–40% for the overall geometry of protein interactions [88]; 50% for quaternary structure [89]; above 65% for protein partner conservation [90]; and between 60–80% for a series of function related properties [91]. That is, there is a sequence identity region between 50% and 70% defining the boundaries of protein function conservation: above this region protein function will be generally conserved, and therefore will be unlikely to contribute to PheDif. On the contrary, below 50% sequence identity protein function will generally vary and therefore will be more likely to contribute to PheDif.

In the case of IMco, we know that its values are obtained for sets of genes (see also *Materials and Methods* and Figure 1). When IMco is equal to 1 all human and mouse orthologs in a set will have IM, consequently there will be no changes in IM contributing to PheDif; if IMco values become smaller, the fraction of ortholog pairs able to establish inter-species PheDif will grow. However, this will only apply when differences in IM involve isoforms that are both functional and species-specific. In the following we comment on these two points. (Note that a different issue, unrelated to IMco values, is when contributions to PheDif result from differences in other gene product properties, like number or nature of isoforms; this is not considered here).

The functionality of gene isoforms and their amount is still an unsolved problem [9], [92], [93]. In our first dataset 86% and 72% of human and mouse genes have IM, respectively (84% for both species, when using dataset 2). These values are high, and in line with those mentioned in the ENCODE project [94] and in recent high-throughput transcriptomics experiments [27], [28], [42], [95] where a large majority of human multi-exon genes (and probably those from other mammal species) is found to have multiple isoforms. Are all these isoforms functional? That is, when expressed do they contribute a new function to the cell proteome or play a regulatory role? Because the experimental approach required to provide an answer for the thousands of known isoforms is so complex, these questions are still open. On one side, studies carried in specific systems [96]–[100] support the functional role of IM. This is also supported by large-scale studies. Using a quantitative microarray Pan et al. [37] established a link between tissue-specific alternative splicing and functional effects; Ellis et al. [101] and Buljan et al. [102] have characterized the relationship between tissue-specific alternative splicing, protein-protein interactions and protein disorder, a result supported by Barbosa-Morais et al. [27]; and Merkin et al. [28] have related alternative splicing and phosphorylatability.

On the other side, a growing amount of data indicates that not all expressed isoforms are functional [9], [92], [93], [103]–[105]. In particular, it has been found that abundance and nature of transcript data are consistent with the existence of noisy splicing [103], [104], [106], [107]. Recently, Pickrell et al. [93] have used RNA-seq to show that indeed an important amount of alternative isoforms result from noisy splicing. On the same line, Hon et al. [92] have used RNA-seq in *E.histolytica* to show that a majority of alternative splicing and polyadenylation isoforms are the result of stochastic processes and therefore unlikely to play a functional role. Reinforcing these results, recent proteomics studies [105] show that a fraction of transcripts do not reach the protein level, and for this reason are less likely to be functional.

We mentioned before that the second requirement that species-specific IM must fulfill to contribute to inter-species PheDif is that it must not correspond to genetically-driven, or individual, IM. The latter will contribute to intra-species, but not to inter-species, differences. At present it is well accepted that this variability exists [108]–[112], although its proportion is yet unknown.

In summary, from the previous considerations it is clear that a certain amount of the isoforms contributing to IMco will have no impact on inter-species PheDif. For this reason, phenotypically relevant IMco values will be lower than those observed in Figure 2.

**Figure 10:**
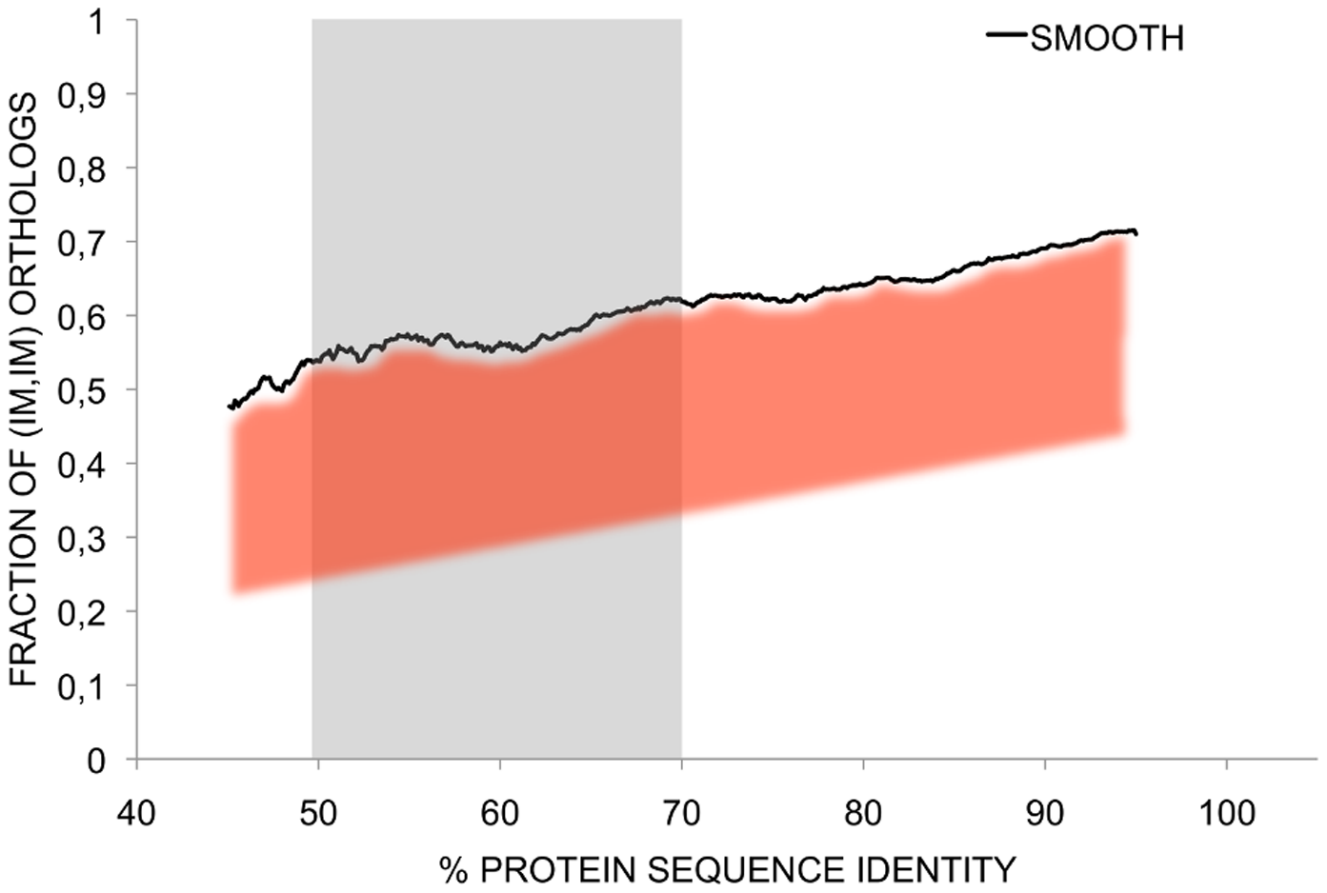
The interplay between isoform multiplicity co-occurrence (IMco) and protein divergence (PD) in the generation of human-mouse phenotypic differences. Here we plot the same graph as in Figure 2A with two additions: a grey-shaded area corresponding to the 50%–70% zone that separates functional from non-functional PD (see *Discussion*); a red-shaded area below the black line, highlighting the fact that the latter is an upper threshold for IMco values obtained after exclusion of non-relevant isoforms. We can see that phenotypically-relevant IMco values (red-shaded area) are always lower than 1, indicating that differences in isoform multiplicity can contribute to human-mouse phenotypic differences all over the PD range. To the right of the grey-shaded area this contribution will be more relevant than that of PD; to the left both phenomena will very likely cooperate to generate phenotypic changes; the situation is unclear within the 50%–70% zone.

We can now go back to our original question: how can we use the IMco-PD relationship to improve our view of the molecular basis of human-mouse PheDif. In Figure 10 we reproduce Figure 2, adding the functional threshold for PD values and highlighting the fact that the present result constitutes an upper threshold for phenotypically relevant IMco. In Figure 10 we highlight in grey the region between 50% and 70%, which corresponds to the protein function threshold (see above). Above this threshold IMco values are already below one, indicating that in general IM differences but not PD will contribute to PheDif between human and mouse.

For the grey region between 50% and 70% sequence identity little can be said. However, below this region the situation is complex, with functional changes resulting from sequence divergence being accompanied by phenotypically-relevant changes in IMco. This points to a rich scenario in which both phenomena would frequently cooperate to originate PheDif between species.

## Conclusions

To understand the interplay between the molecular contributors to PheDif we studied the relationship between IMco and PD, using three sets of human-mouse orthologs. We found that there was a monotonic dependence between IMco and PD that could be traced to a more fundamental relationship: the link between exon number of the longest gene isoform and PD. Given its interest, we explored the existence of this second relationship in other species, finding that this was the case, although with variations in the monotonic behavior (for the fruit fly case). Using previous results from the literature we could provide a plausible explanation for this relationship in terms of the balance between functional and cost of synthesis constraints. Finally, we show how the IMco-PD relationship could be used to analyze the molecular basis of inter-species PheDif.

## Supporting Information

Figure S1 **Relationship between isoform multiplicity co-occurrence (IMco) and protein divergence (PD).** This figure is equivalent to Figure 2, with the difference that IMco estimates obtained from less than 5 ortholog pairs have been eliminated. (A) and (B) correspond to Ensembl and VEGA data. In both figures black dots are used to show the raw, unprocessed data; a blue line is used for the smoothed data, which is shown with its envelope in grey.
(TIF)

Figure S2 **The contribution of species-specific isoform multiplicity (IM) to isoform multiplicity co-occurrence (IMco) (VEGA data).** This figure is equivalent to Figure 3, except in that we have used VEGA, instead of Ensembl, isoform data. Here we compare IMco with the product of species-specific IM, P(IM_H_|x)⋅P(IM_M_|x), a term of IMco, as shown in Equation 2 (see *Materials and Methods*). In (A) we show the raw data representation: we can observe an important overlap between both data clouds, as well as a similar monotonic trend, something confirmed in (B) where we show the smoothed data. In (A) the color code is: black for IMco and grey for P(IM_H_|x)⋅P(IM_M_|x); in (B) we have dark blue for IMco, and light blue for P(IM_H_|x)⋅P(IM_M_|x).
(TIF)

Figure S3 **Species-specific isoform mutiplicity (IM) vs. protein divergence (PD) (VEGA data).** This figure is equivalent to Figure 4, except in that we have used VEGA, instead of Ensembl, isoform data. We show the relationship between species-specific IM and PD, for both human and mouse genes. In both (A) and (B) we plot raw data, with black black and grey dots for human and mouse, respectively. Also, in both (A) and (B) we plot a smoothed version of these raw data: yellow and red for human and mouse, respectively, and grey for the corresponding envelopes. Finally, (A) differs from (B) in that for the latter we have eliminated those estimates of IMco obtained from less than 5 observations. We observe the same monotonically increasing trend for both species, a result that provides a simple explanation for the also monotonically increasing behavior of P(IM_H_|x)⋅P(IM_M_|x) (Figures 3 and S2), the product of species-specific IM.
(TIF)

Figure S4 **Mouse-specific isoform mutiplicity (IM) vs. protein divergence (PD) (RNA-seq data).** This figure is equivalent to Figures 4 and S3, except in that we have restricted our analysis to the mouse case and used RNA-seq, instead of Ensembl, isoform annotations (data for three individuals from Merkin et al. [28]). We show the relationship between species-specific IM and PD for mouse genes, under scenarios that combine different quality controls for RNA-seq data (mild, FPKM>0; less permissive, FPKM≥1) and the effect of individual variability in RNA-seq data: (A) lax quality control, individual variability ignored (results from three samples considered together); (B) lax quality control, individual variability considered (to annotate a given gene as having IM, it had to display IM in at least one tissue for each of three individuals); (C) less permissive quality control, individual variability ignored; and (D) less permissive quality control, individual variability considered. In all the plots we have two curves: in green we have the results obtained with RNA-seq data only, in brown we have the results obtained with RNA-seq data enriched with Ensembl annotations (as a result more genes are annotated as having IM). Dots represent the raw data and continuous lines are used for the smoothed data, which are shown with the corresponding envelope in grey. As a minimum quality control, IM is estimated when more than 5 observations are available. In all four plots we observe a monotonically increasing trend between mouse IM and PD (Spearman rank correlation, rho, and p-values from Figures S4A to S4D: rho = 0.15, p-val = 0.002; rho = 0.22, p-val = 1.9×10^−6^; rho = 0.28, p-val = 2.×8^−^10^−9^; rho = 0.36, p-val = 1.3×10^−14^), in accordance with the results found in Figures 4 and S3.
(TIF)

Figure S5 **Number of exons vs. protein divergence (PD) (VEGA data).** This figure is equivalent to Figure 6, except in that we have used VEGA, instead of Ensembl, isoform data. We show the relationship between number of exons of the largest isoform and PD, for both human and mouse genes. In both (A) and (B) we plot raw data, with black and grey dots for human and mouse, respectively. Also, in both (A) and (B) we plot a smoothed version of these raw data: yellow and red for human and mouse, respectively, and grey for the corresponding envelopes. Finally, (A) differs from (B) in that for the latter we have eliminated those estimates of IMco obtained from less than 5 observations.
(TIF)
