# Supplementary figures and images for: The Relationship between Gene Isoform Multiplicity, Number of Exons and Protein Divergence
Source: PLoS One. 2013 Aug 30;8(8):e72742. doi: 10.1371/journal.pone.0072742 (PMC3758341; doi:10.1371/journal.pone.0072742)

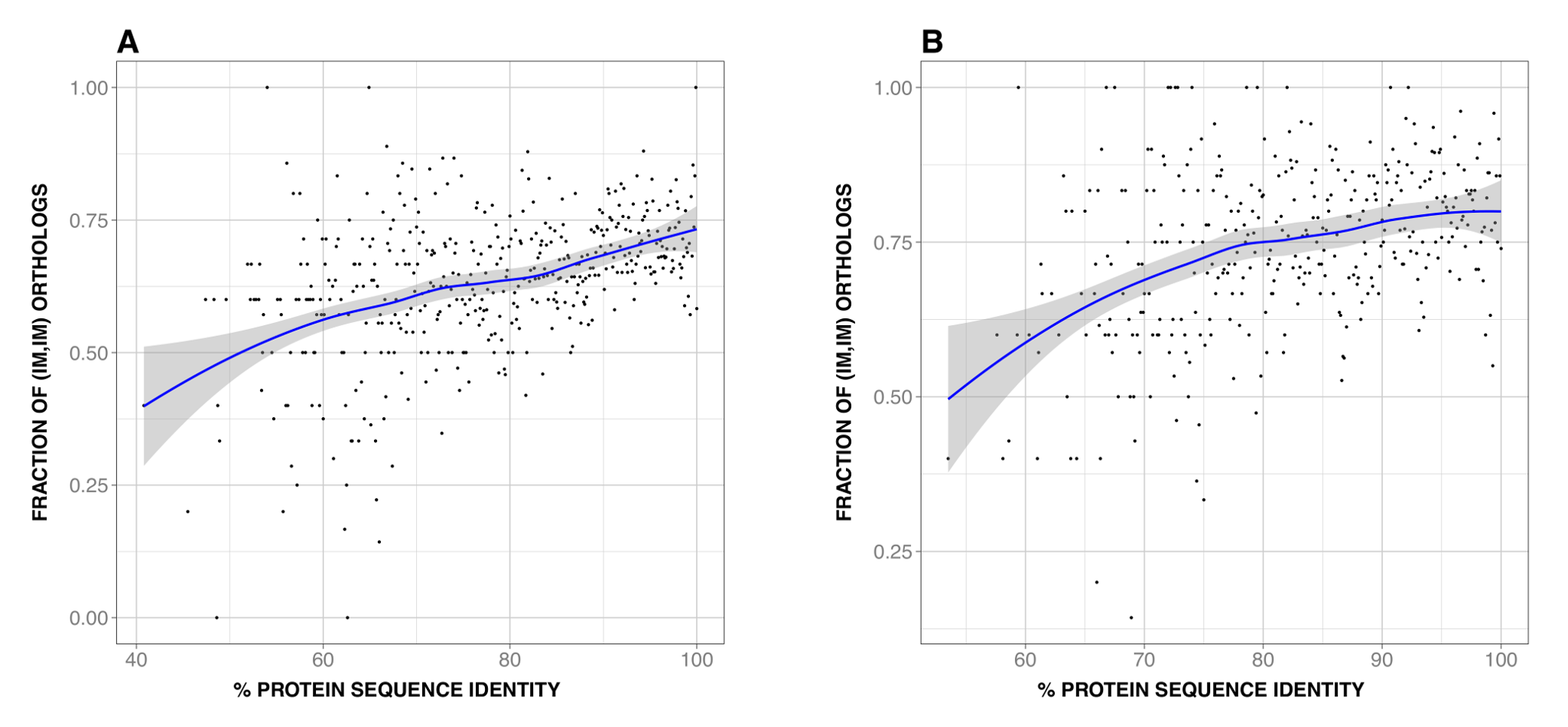

Supplement: Figure S1 — Relationship between isoform multiplicity co-occurrence (IMco) and protein divergence (PD). This figure is equivalent to Figure 2, with the difference that IMco estimates obtained from less than 5 ortholog pairs have been eliminated. (A) and (B) correspond to Ensembl and VEGA data. In both figures black dots are used to show the raw, unprocessed data; a blue line is used for the smoothed data, which is shown with its envelope in grey. (TIF) [file pone.0072742.s001.tif]

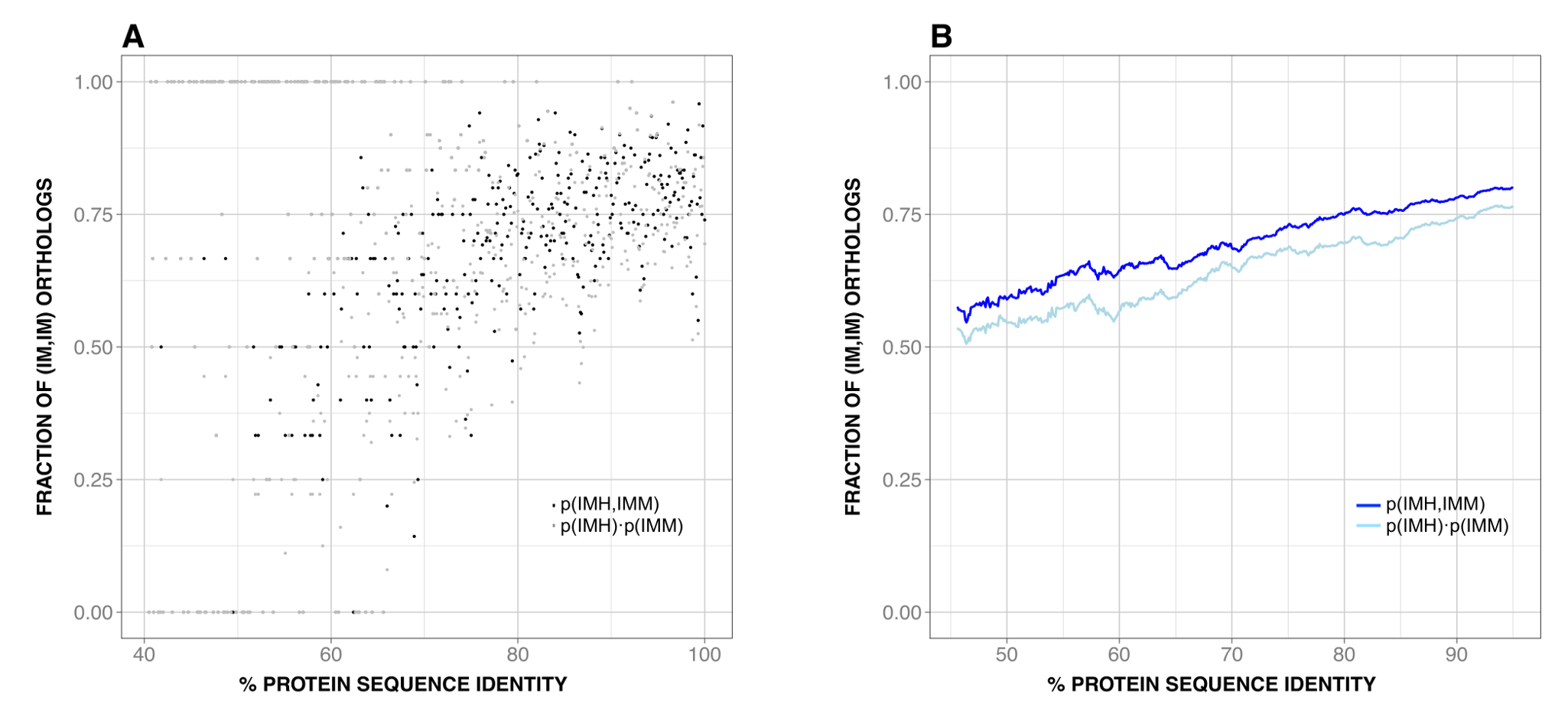

Supplement: Figure S2 — The contribution of species-specific isoform multiplicity (IM) to isoform multiplicity co-occurrence (IMco) (VEGA data). This figure is equivalent to Figure 3, except in that we have used VEGA, instead of Ensembl, isoform data. Here we compare IMco with the product of species-specific IM, P(IMH|x)⋅P(IMM|x), a term of IMco, as shown in Equation 2 (see Materials and Methods). In (A) we show the raw data representation: we can observe an important overlap between both data clouds, as well as a similar monotonic trend, something confirmed in (B) where we show the smoothed data. In (A) the color code is: black for IMco and grey for P(IMH|x)⋅P(IMM|x); in (B) we have dark blue for IMco, and light blue for P(IMH|x)⋅P(IMM|x). (TIF) [file pone.0072742.s002.tif]

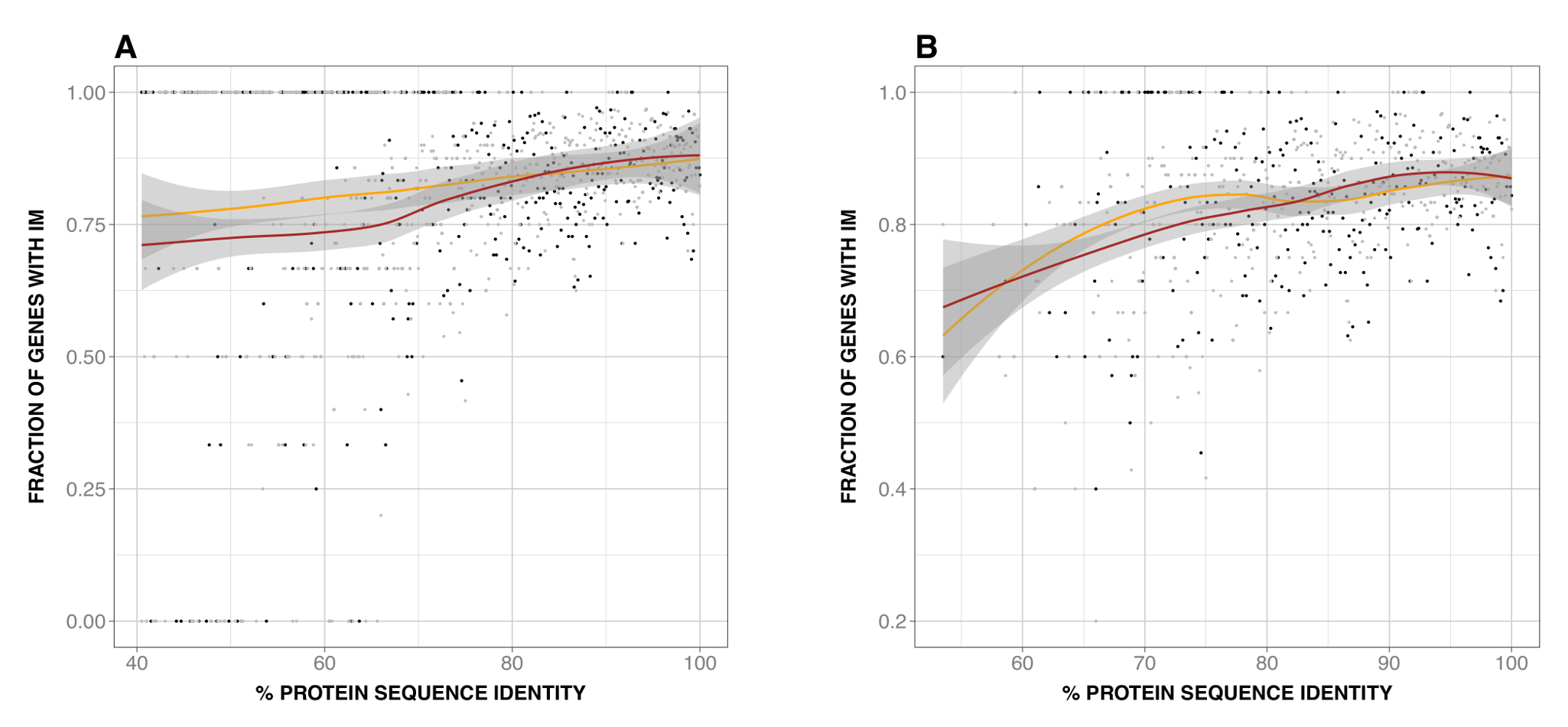

Supplement: Figure S3 — Species-specific isoform mutiplicity (IM) vs. protein divergence (PD) (VEGA data). This figure is equivalent to Figure 4, except in that we have used VEGA, instead of Ensembl, isoform data. We show the relationship between species-specific IM and PD, for both human and mouse genes. In both (A) and (B) we plot raw data, with black black and grey dots for human and mouse, respectively. Also, in both (A) and (B) we plot a smoothed version of these raw data: yellow and red for human and mouse, respectively, and grey for the corresponding envelopes. Finally, (A) differs from (B) in that for the latter we have eliminated those estimates of IMco obtained from less than 5 observations. We observe the same monotonically increasing trend for both species, a result that provides a simple explanation for the also monotonically increasing behavior of P(IMH|x)⋅P(IMM|x) (Figures 3 and S2), the product of species-specific IM. (TIF) [file pone.0072742.s003.tif]

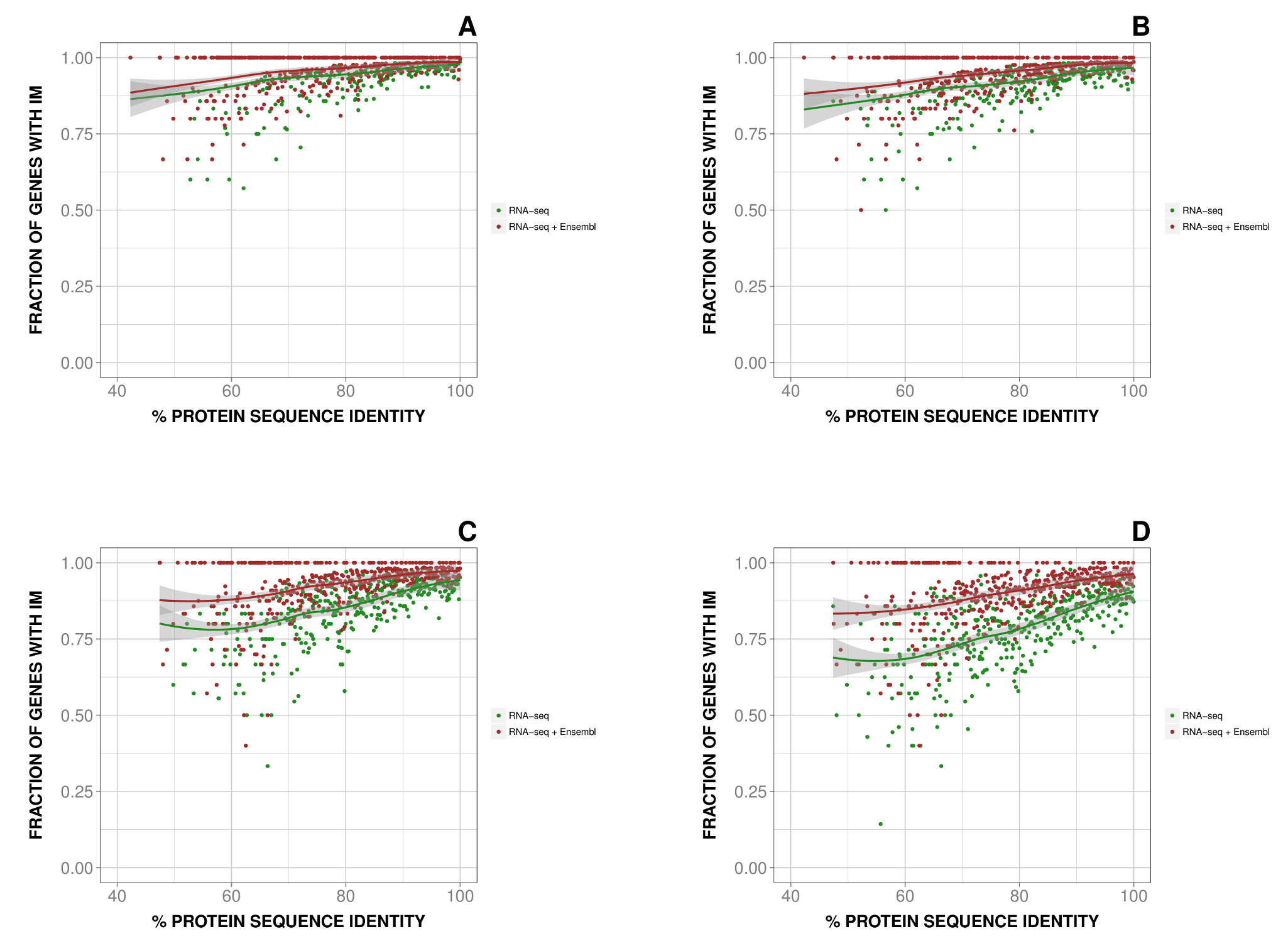

Supplement: Figure S4 — Mouse-specific isoform mutiplicity (IM) vs. protein divergence (PD) (RNA-seq data). This figure is equivalent to Figures 4 and S3, except in that we have restricted our analysis to the mouse case and used RNA-seq, instead of Ensembl, isoform annotations (data for three individuals from Merkin et al. [28]). We show the relationship between species-specific IM and PD for mouse genes, under scenarios that combine different quality controls for RNA-seq data (mild, FPKM>0; less permissive, FPKM≥1) and the effect of individual variability in RNA-seq data: (A) lax quality control, individual variability ignored (results from three samples considered together); (B) lax quality control, individual variability considered (to annotate a given gene as having IM, it had to display IM in at least one tissue for each of three individuals); (C) less permissive quality control, individual variability ignored; and (D) less permissive quality control, individual variability considered. In all the plots we have two curves: in green we have the results obtained with RNA-seq data only, in brown we have the results obtained with RNA-seq data enriched with Ensembl annotations (as a result more genes are annotated as having IM). Dots represent the raw data and continuous lines are used for the smoothed data, which are shown with the corresponding envelope in grey. As a minimum quality control, IM is estimated when more than 5 observations are available. In all four plots we observe a monotonically increasing trend between mouse IM and PD (Spearman rank correlation, rho, and p-values from Figures S4A to S4D: rho = 0.15, p-val = 0.002; rho = 0.22, p-val = 1.9×10^−6^; rho = 0.28, p-val = 2.×8−10−9; rho = 0.36, p-val = 1.3×10^−14^), in accordance with the results found in Figures 4 and S3. (TIF) [file pone.0072742.s004.tif]

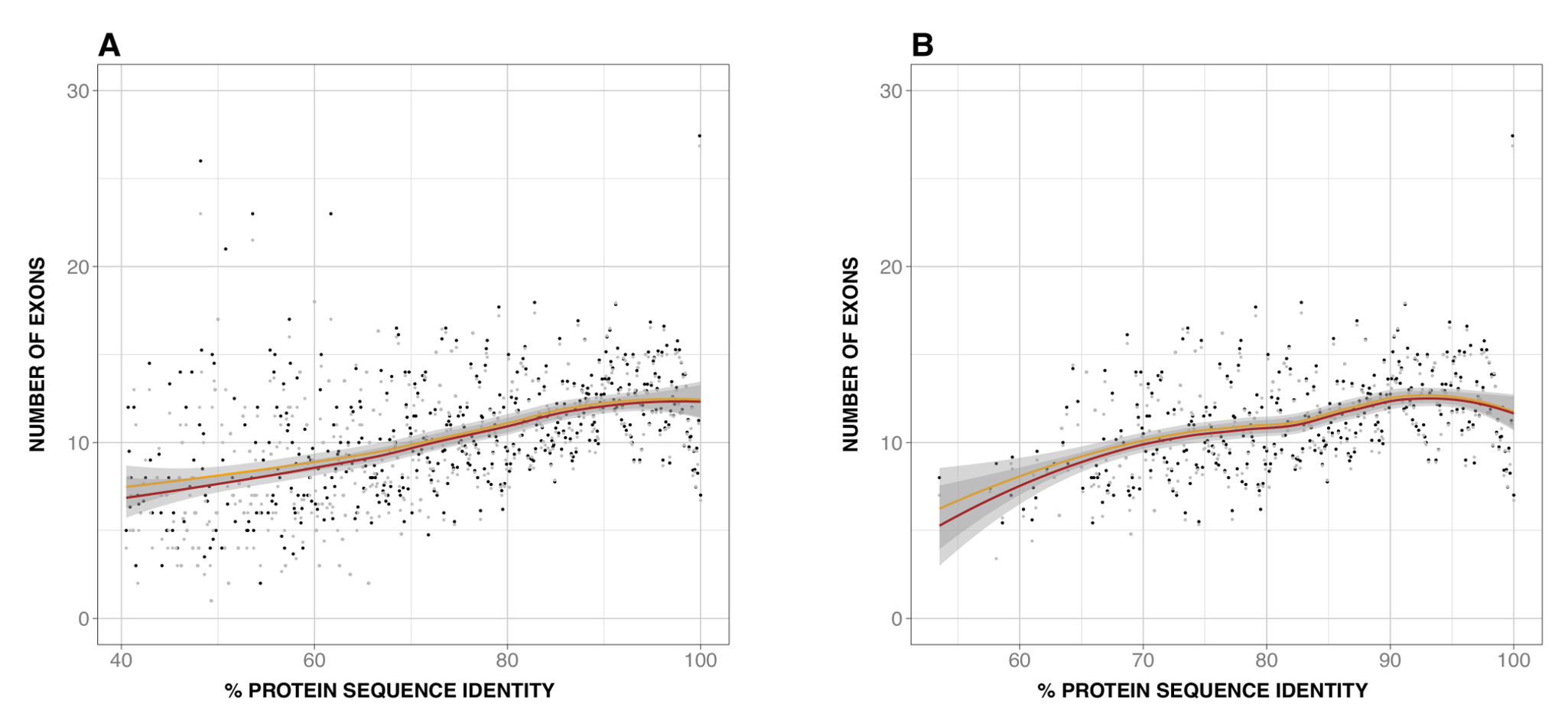

Supplement: Figure S5 — Number of exons vs. protein divergence (PD) (VEGA data). This figure is equivalent to Figure 6, except in that we have used VEGA, instead of Ensembl, isoform data. We show the relationship between number of exons of the largest isoform and PD, for both human and mouse genes. In both (A) and (B) we plot raw data, with black and grey dots for human and mouse, respectively. Also, in both (A) and (B) we plot a smoothed version of these raw data: yellow and red for human and mouse, respectively, and grey for the corresponding envelopes. Finally, (A) differs from (B) in that for the latter we have eliminated those estimates of IMco obtained from less than 5 observations. (TIF) [file pone.0072742.s005.tif]
